# Insight into the significance of Foxp3 + tumor-infiltrating lymphocytes in squamous cell lung cancer

**DOI:** 10.1007/s12094-024-03392-w

**Published:** 2024-02-25

**Authors:** Kazu Shiomi, Masaaki Ichinoe, Ai Ushiwata, Koji Eshima, Ryo Nagashio, Shoko Hayashi, Dai Sonoda, Yasuto Kondo, Raito Maruyama, Masashi Mikubo, Yoshiki Murakumo, Yukitoshi Satoh

**Affiliations:** 1 Department of Thoracic Surgery, Kitasato University School of Medicine, 1-15-1 Kitasato, Minami-Ku, Sagamihara-Shi, Kanagawa, 252-0374 Japan; 2 Department of Pathology, Kitasato University School of Medicine, 1-15-1 Kitasato, Minami-Ku, Sagamihara-Shi, Kanagawa, 252-0374 Japan; 3 Department of Clinical Medicine (Biostatistics), Kitasato University School of Pharmacy, 5-9-1 Shirokane, Minato-Ku, Tokyo, 108-8641 Japan; 4 Department of Biosciences, Kitasato University School of Sciences, 1-15-1 Kitasato, Minami-Ku, Sagamihara-Shi, Kanagawa, 252-0374 Japan; 5 Department of Applied Tumor Pathology, Graduate School of Medical Sciences, Kitasato University, 1-15-1 Kitasato, Minami-Ku, Sagamihara-Shi, Kanagawa, 252-0374 Japan

**Keywords:** Biomarker, Tumor-infiltrating lymphocytes, Foxp3, Regulatory T cells, Lung cancer

## Abstract

**Purpose:**

Although developing a better understanding of tumor-infiltrating Foxp3 + lymphocytes (Foxp3 + TILs) might provide essential knowledge to predict response to immunotherapy and prognosis, our current knowledge about Foxp3 + TILs is inadequate. This study investigated the prognostic significance of tumor-infiltrating Foxp3 + lymphocytes (Foxp3 + TILs) in squamous cell lung cancer (SQ-LC) objectively.

**Methods:**

Among patients with SQ-LC surgically resected in our institution between 2011 and 2017, those with pathological stage IA3-IIIA were immunohistochemically studied to evaluate Foxp3 + TILs in their tumor stroma. The impact of Foxp3 + TILs on relapse-free survival (RFS) was analyzed with Kaplan–Meier survival analysis and multivariate analysis using a Cox proportional hazards model/Fine-Gray model.

**Results:**

This study analyzed 100 patients. Multivariate analysis showed that a large number of Foxp3 + TILs in the stroma does not associate with a poor prognosis, rather that a large number of Foxp3 + TILs (≥ 64 cells) tend to be associated with a more favorable prognosis than a small number of Foxp3 + TILs (< 64 cells) (large vs small number: HR, 0.56; 95% CI, 0.17–1.83; *P* = 0.34). Exploratory analysis also showed that in the two populations divided by a difference in Foxp3 expression levels, similar trends to the main analysis were observed.

**Conclusion:**

Our results showed that a large number of Foxp3 + TILs in the stroma may not associate with a poor prognosis in SQ-LC. To use the seemingly complicated information of Foxp3 + TILs as biomarkers, better understanding the diversity and heterogeneity of Foxp3 + TILs and analyzing their subpopulations that increase in the TME may be needed.

## Introduction

Although immune checkpoint inhibitors have been proven to improve survival in a variety of cancer types, only a subset of patients derive true benefit in clinical practice [1]. Maximizing the potential of immune checkpoint inhibitors requires refined biomarkers to predict prognosis, efficacy, and immune-related adverse events. Among various approaches used in biomarker discovery, an approach that tries to find clues from a deep understanding of the tumor microenvironment (TME), and especially the status of tumor-infiltrating lymphocytes (TILs), is thought to be attractive [2, 3] because the immune cell phenotypes in the TME may provide information on the mechanisms of immune evasion and the current situation of tumor-immune interactions.

Of the TILs, regulatory T (Treg) cells, which are a subset of CD4 + T cells characterized by expression of the transcription factor Foxp3 in the nucleus and CD25 and CTLA-4 on the cell surface, are generally thought to be one of the key players inhibiting antitumor immunity [4–6]. Therefore, the research for TILs focusing on the Foxp3 molecule, which is thought to be expressed specifically in Tregs, have considered attractive in predicting the degree of poor prognosis. However, in fact, the results among studies are inconsistent, and the significance of Foxp3 + TILs as a biomarker has not been established [7–13]. Moreover, there are also reports stating that when inflammation occurs, T cells other than Tregs can express Foxp3, or the function of Tregs, which originally express Foxp3, changes (although this has not been clearly proven), such that the components of Foxp3 + TILs become heterogeneous [14–16].

In lung cancer, there are only a few reports showing Foxp3 + TILs as an unfavorable prognostic factor, and they have focused mainly on adenocarcinoma [17–21]. Hence, in the present study, we investigated the prognostic significance of Foxp3 + TILs in a more objective way by focusing on squamous cell lung cancer (SQ-LC), which is often accompanied by inflammation in the TME.

## Material and method

### Patients and clinical samples

This retrospective cohort study was approved by the Kitasato University Medical Ethics Committee (approval no. B17-213). All patients who underwent complete resection of SQ-LC at our institution between January 1, 2011 and December 31, 2017 were identified from the database of the Thoracic Surgery Department. Subsequently, we selected patients with pathological stage IA3 to IIIA according to the 8th edition of the TNM classification. Exclusion criteria included (1) radiotherapy and/or chemotherapy before surgery, (2) inadequate tissue slice, (3) multiple primary lung cancers with advanced adenocarcinoma, (4) death from early postoperative complications, and (5) wedge resection.

### Immunohistochemical analysis

For our analysis, we chose one whole tissue slide from each patient that contained the border of the tumor and normal lung tissue, as many lymphocytes as possible, and the least amount of necrosis. Tissue Sects. (3-µm) prepared from formalin-fixed, paraffin-embedded tissue were immunostained using the Bond-MAX Automated Immunohistochemistry system and Bond Polymer Refine Detection Kit (DC 9800; Leica Biosystems, Newcastle, UK). The approximately 3-h protocol includes the following steps: sections were deparaffinized and pretreated with Bond Epitope Retrieval Solution 2 (ER2, EDTA-based buffer, pH 9.0; Leica Biosystems) at 100 °C for 20 min. After washing, peroxidase blocking was carried out for 10 min. Tissues were again washed and then incubated with 200 × diluted anti-Foxp3 mouse monoclonal antibody (clone: 236A/E7, Abcam) for 30 min. Finally, the sections were incubated with Bond Polymer (Leica Biosystems) for 10 min, developed with DAB-chromogen for 10 min and counterstained with hematoxylin for 5 min.

For each studied case, the four areas bordering the cancer cell nest that were quantitatively the richest in Foxp3 + TILs in the tumor stroma were carefully selected. Peribronchial and perivascular areas, where lymphocyte aggregates are normally observed, and lymphoid follicles were excluded from the selection.

The number of Foxp3 + TILs in these “hot spot” areas per high-power field (400 ×) were calculated by e-Count cell counting software (e-path; Fujisawa, Kanagawa, Japan), which is an automatic measuring instrument, and then checked visually to make corrections. Then, the average values of the four fields were analyzed statistically.

### Statistical analysis

The minimum p-value method was applied to determine the optimal cut-off value of the averaged number of Foxp3 + TILs. The primary endpoint of this study was relapse-free survival (RFS), which was defined as the interval between the date of surgery to the date of proven recurrence or death from any cause. Data for patients lost to follow-up were censored on the date the patient was last seen alive without recurrence. Cumulative survival curves were estimated with the Kaplan–Meier method and compared with the log-rank test and Wilcoxon signed rank test. Multivariable analysis was performed with the Cox proportional hazards regression model and the Fine-Gray model to estimate the independent prognostic effect on RFS of the number of Foxp3 + TILs in tumor stroma by adjusting for confounding factors [22]. Variables were selected for multivariate analysis on the basis of the results of the univariate analysis, statistical independence, number of events, and clinical significance for postoperative recurrence. In this study, we performed multivariate analysis considering the risk of competition. Because of our higher interest in cancer-specific recurrence, we defined this as an event of interest and death from other diseases and death without evidence of relapse as competing risks.

We thought it would be appropriate to use the above method to determine the relationship between prognostic factors and RFS more specifically, especially in the situation where it cannot be denied that deaths from other diseases affect the probability of achieving RFS, because of the high ratio of deaths from other diseases to the total number of RFS events (about 22%). A *P* value of < 0.05 was considered significant. All analyses were conducted with JMP Pro version 17.1 (SAS Inc., Cary, NC), EZR (Saitama Medical Center, Jichi Medical University; http://www.jichi.ac.jp/saitama-sct/SaitamaHP.files/statmed.html; Kanda), and R version 4.1.2.

## Results

### Patient characteristics

During the study period, 113 patients underwent complete resection for pathological stage IA3 to IIIA SQ-LC. Excluded were 3 patients with radiotherapy or chemotherapy before surgery, 3 patients with inadequate tissue slice, 2 patients with multiple primary lung cancers with advanced adenocarcinoma, 1 patient with death from early postoperative complications, and 4 patients with wedge resection. Finally, 100 patients remained and were analyzed in this study.

The age of these patients ranged between 44 and 84 with an average age of 72 years. Among them, 87% were male, 59% had stage II or III disease, and 22% received postoperative adjuvant chemotherapy.

At the time of analysis, 24 patients had died of the disease, 14 had died of other causes, and 38 had experienced recurrence. The median duration of follow-up was 55 months from the pulmonary resection.

### Evaluation of Foxp3 + TILs

The numbers of Foxp3 + TILs in the stroma ranged between 5.8 and 120.8 (median, 27.8; interquartile range, 19.1–42.4). The cut-off value of the averaged number of Foxp3 + TILs was 64 via the minimum *p*-value method.

**Fig. 1:**
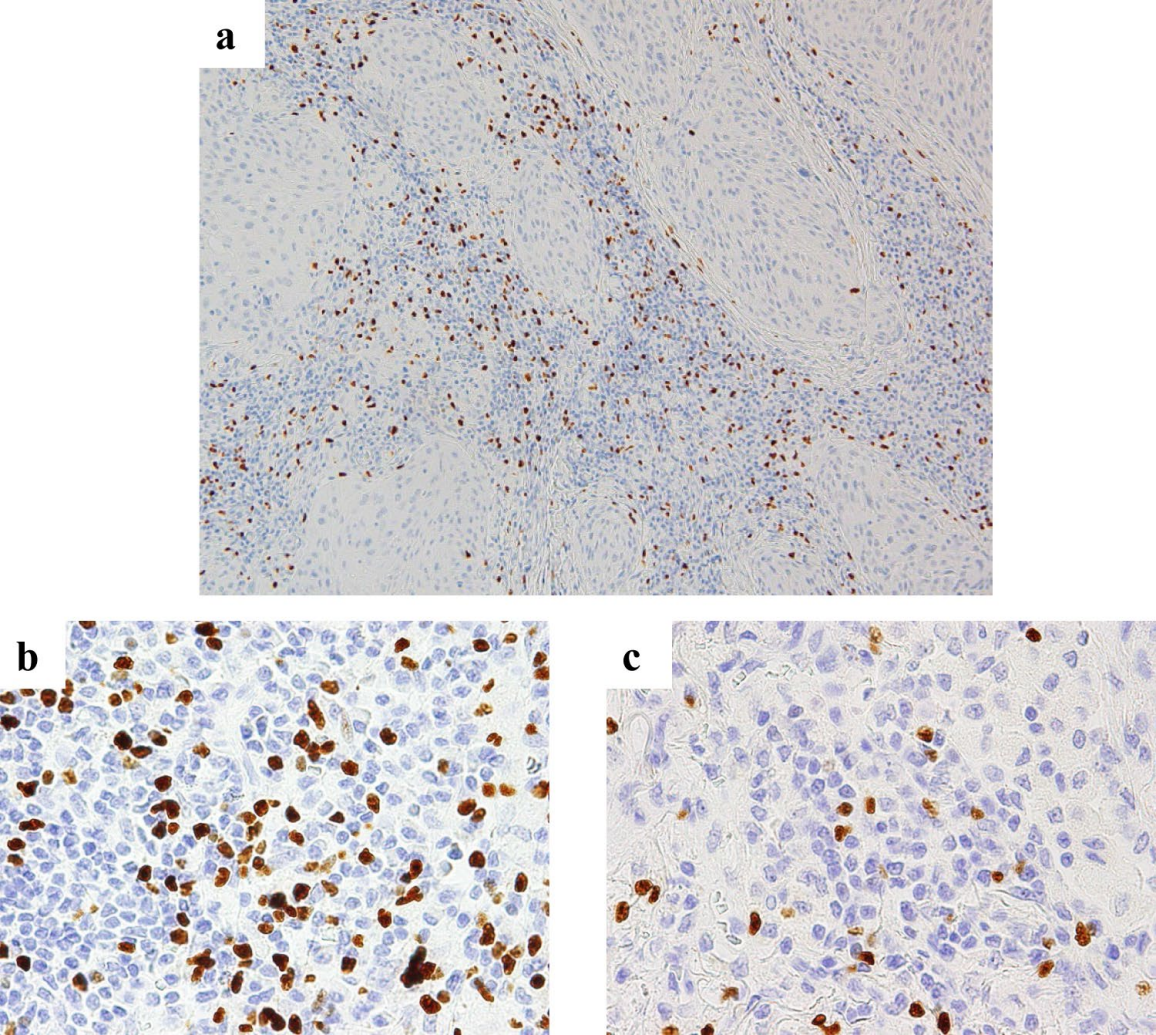
Histopathological images. **a** Foxp3 + TILs in the tumor stroma (magnification × 100). **b** Image showing a large number of Foxp3 + TILs, and **c** image showing a small number of Foxp3 + TILs (magnification × 400). *Foxp3* + *TILs* tumor-infiltrating Foxp3 + lymphocytes

Figure 1 shows representative histopathological images of Foxp3 + TILs in the stroma for low-power field imaging and each category (magnification: a × 100; b, c × 400). The relationships between the numbers of Foxp3 + TILs and the clinicopathological characteristics are summarized in Table 1. There was no significant correlation between the numbers of Foxp3 + TILs in tumor stroma and any of the other factors shown in Table 1.

**Table 1:** Patient characteristics and association between the number of tumor-infiltrating Foxp3 + lymphocytes in the tumor stroma and clinicopathological parameters

Variable	n	Number of Foxp3 + TILs		*P* value
		Large (≥ 64)	Small (< 64)	
*Age *(*years*)				
≥ 75	30	2 (7)	28 (93)	0.283
< 75	70	10 (14)	60 (86)	
*Sex*				
Male	87	10 (11)	77 (89)	0.687
Female	13	2 (15)	11 (84)	
*Pathological stage*				
I (IA3/IB)	41	5 (12)	36 (88)	0.938
II	30	4 (13)	26 (87)	
III	29	3 (10)	26 (90)	
*Tumor size, cm*				
≤ 3.0	39	5 (13)	34 (87)	0.840
> 3.0	61	7 (11)	54 (89)	
*Nodal status*				
N0	67	7 (10)	60 (90)	0.496
N1/N2	33	5 (15)	28 (85)	
*Vascular invasion*				
No	26	1 (4)	25 (96)	0.137
Yes	74	11 (15)	63 (85)	
*Lymphatic invasion*				
No	60	8 (13)	52 (87)	0.615
Yes	40	4 (10)	36 (90)	
*Pleural invasion*				
No	54	7 (13)	47 (87)	0.748
Yes	46	5 (11)	41 (89)	
*Adjuvant chemotherapy*				
No	78	8 (10)	70 (90)	0.312
Yes	22	4 (18)	18 (82)	

*Foxp3 TILs* tumor-infiltrating Foxp3 + lymphocytes

Values are *n* (%) unless otherwise noted

### Survival analysis

**Fig. 2:**
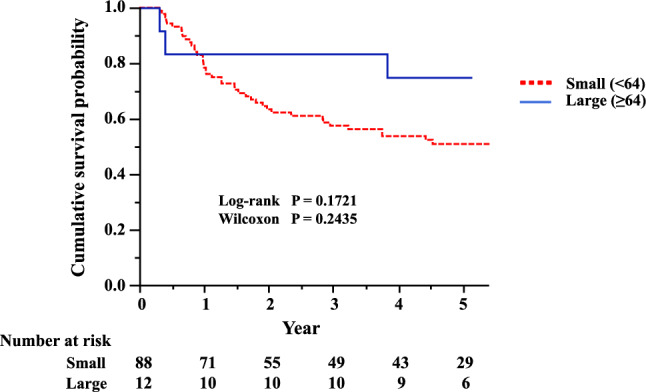
Relapse-free survival curves of patients according to the number of Foxp3 + TILs in the tumor stroma. *Foxp3* + *TILs* tumor-infiltrating Foxp3 + lymphocytes

There was no statistical difference in cumulative RFS survival curves between a large number of Foxp3 + TILs (≥ 64 cells) and a small number of Foxp3 + TILs (< 64 cells) (Fig. 2). However, the differences in the cumulative survival curves rather showed that a large number of Foxp3 + TILs (≥ 64 cells) tended to be associated with a more favorable prognosis than a small number of Foxp3 + TILs (< 64 cells).

The results of multivariate analysis with the Cox proportional hazards regression model were consistent with those of the univariate analysis (Table 2). Similar results were also obtained by multivariate analysis with the Fine-Gray model (large number group vs small number group in the stroma: hazard ratio, 0.84; 95% confidence interval, 0.24–2.92; *P* = 0.780). With regard to the effects of the other clinicopathologic variables on RFS, the directions of the hazard ratios were reasonable.

**Table 2:** Multivariate analysis for relapse-free survival

Variables	HR	95% CI	*P* value
Age (≥ 75/ < 75 years)	1.54	0.82–2.91	0.178
Sex (M/F)	1.84	0.63–5.31	0.263
Pathological stage (III/I, II)	1.54	0.79–3.00	0.204
Adjuvant chemotherapy (Yes/No)	0.62	0.25–1.54	0.306
Foxp3 + TILs in the tumor stroma (≥ 64/ < 64)	0.56	0.17–1.83	0.335

*HR* hazard ratio, *CI* confidence interval, *TILs* tumor-infiltrating lymphocytes

### Exploratory analysis

**Fig. 3:**
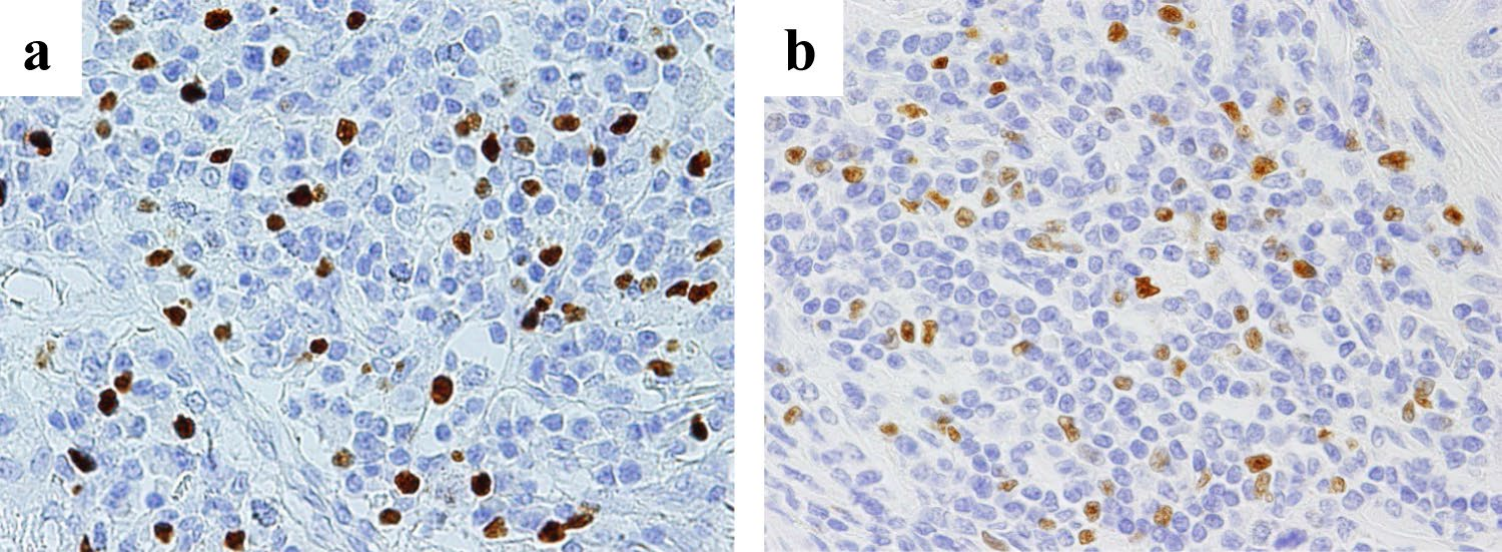
Histopathological images (magnification × 400) for each category. **a** High expression group: the entire nucleus is strongly and uniformly stained. **b** Low expression group: nuclear staining is weak but nucleoli are recognizable

**Fig. 4:**
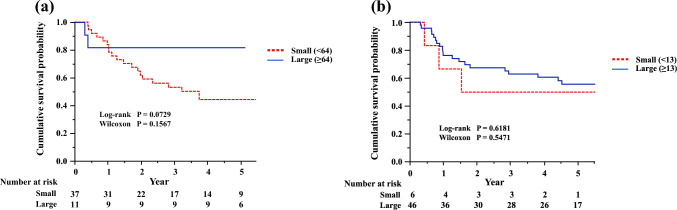
**a** RFS curves of patients according to the number of Foxp3 + TILs in the tumor stroma in the high expression group. **b** RFS curves of patients according to the number of Foxp3 + TILs in the tumor stroma in the low expression group. *Foxp3* + *TILs* tumor-infiltrating Foxp3 + lymphocytes, *RFS* relapse-free survival

We divided Foxp3 + TILs into two groups, a high expression group and a low expression group based on the difference in their expression levels and analyzed whether the clinical significance of Foxp3 + TILs in each group differed (Fig. 3a, b). Similar trends to the main analysis were also observed in the two groups (Fig. 4a, b).

## Discussion

Treg cells have been reported to suppress autoimmunity and excessive inflammation by suppressing self-reactive T cells and inflammatory immune cells [23, 24]. Also in regard to tumor immunity, several preclinical and clinical studies have suggested that Foxp3 + Treg cells suppress anti-tumor immune responses [5, 6, 25, 26]. In the past, Foxp3 has been thought to be a master regulatory gene specifically expressed in Treg cells, and Foxp3 + TILs have been regarded as being nearly identical to Treg cells. In fact, there have been reports that high infiltration of Foxp3 + TILs in the TME is associated with an unfavorable prognosis in patients with many types of cancers [7–10]. Recently, however, various other functions and roles of Foxp3 + Treg cells, which include tissue repair, organismal metabolism, and neuronal pruning in preserving tissue homeostasis, have been reported [27, 28]. Furthermore, also relating to tumor immunity, several reports have shown that there are some subpopulations of Foxp3 + T cells with different functions, including non-Treg cells without an immunosuppressive function [15, 26, 29]. There are also reports that Foxp3 + T cells are readily induced from conventional CD4 + T cells in the presence of inflammation [16, 30, 31]. These data indicate the diversity of Foxp3-positive T cells including Treg cells. In fact, for Foxp3 + TILs, there are some conflicting results regarding prognostic significance between patients with colorectal and head and neck cancer and those with melanoma, breast, ovarian, and gastric cancer [11–13, 32]. Putting these facts together, we propose that the significance of Foxp3 + TILs as a prognostic factor in the TME is not as simple as widely believed but may vary depending on the situation of TME.

For lung cancer, there are only a few reports showing that the density of Foxp3 + TILs is associated with poor prognosis [17–21, 33]. Furthermore, the subjects of these studies have either been adenocarcinoma alone or more adenocarcinoma than other histological types of cancer. There are no reports focusing on squamous cell carcinoma, which is different from adenocarcinoma in terms of it being frequently accompanied by inflammation. Therefore, in this study, we investigated the clinical significance of Foxp3 + TILs in SQ-LC by a more objective method using digital pathology scoring software.

Our results showed that a large number of Foxp3 + TILs in the stroma may not be associated with a poor prognosis in SQ-LC. The differences in the cumulative RFS survival curves rather showed that a large number of Foxp3 + TILs (≥ 64 cells) tended to be associated with a more favorable prognosis than a small number of Foxp3 + TILs (< 64 cells).

These results are the opposite of those in many previous reports on various cancer types including lung cancer but are the same as those in several reports on colorectal and head and neck cancers.

We consider our results to be very important and reliable because several papers have recently suggested the heterogeneity and diversity of Foxp3 + CD4 + T cells [15, 16, 26–29, 31]. Sakaguchi et al. showed that in colorectal cancer, there are two subpopulations of Foxp3 + TILs, one of which is characterized by low Foxp3 expression and the inability to inhibit antitumor immunity, and the other by a high Foxp3-expressing population with the ability to inhibit antitumor immunity [23]. Furthermore, they showed that in the inflammatory environment generated by the infiltration of gut bacteria into the TME, the number of T cells with low Foxp3 expression increases and their abundance becomes a favorable prognostic factor, opposite to that in patients with many high Foxp3-expressing T cells in their tumor.

There were also some reports on the properties of Treg cells changing to a Th2-like or Th17-like phenotype in an inflammatory environment [16, 30, 31]. Moreover, Miyao et al. reported that Foxp3 + T cells, in addition to Treg cells, contain a small number of non-Treg cells that transiently express Foxp3 and that these accumulate in the environment of inflammatory cytokines [14]. In addition, several papers have also shown that conventional CD4 + T cells can transiently express Foxp3 upon stimulation [34, 35].

When the above is considered, the difference between our results and those of Foxp3 studies for other cancers might be because we focused our study on SQ-LC, which is often associated with necrosis and inflammation. Likewise, this inflammatory environment in the TME might be the reason for our results being similar to those of some reports on colorectal and head and neck cancers. In any case, at present, it is difficult to use Foxp3 + TILs as a biomarker as it is necessary to better understand which subpopulations among Foxp3 + TILs are increased in each of the inflammatory phenotypes of the TME and their role and relationship to prognosis.

Limitations in our study include all of the biases typically caused by a retrospective design and a single-institution study. Furthermore, there might have been variability in the selection of measurement sites in patients with low stromal area. However, judging from the more objective study method of the present study using whole slides, digital software for counting, and measurement of 4 hot spots, and, in addition, the tendency of the hazard ratio for age, sex, pathological stage, and adjuvant chemotherapy in the multivariate analysis, we are convinced that the results of our study are robust.

The next step of our investigation will include clarification of the diversity and heterogeneity of Foxp3 + TILs in the TME and examination of the relationship between such information and the prognosis and efficacy of immune checkpoint inhibitors.

## Conclusion

Our results showed that a large number of Foxp3 + TILs in the stroma may not associate with a poor prognosis in SQ-LC. To use the seemingly complicated information of Foxp3 + TILs as biomarkers, better understanding the diversity and heterogeneity of Foxp3 + TILs and analyzing their subpopulations that increase in the TME may be needed.

## Data Availability

Raw data were generated at Kitasato University School of Medicine. Derived data supporting the findings of this study are available from the corresponding author on request.
